# Psychometric evaluation and updated community norms of the WHO-5 well-being index, based on a representative German sample

**DOI:** 10.3389/fpsyg.2025.1592614

**Published:** 2025-07-29

**Authors:** Sören Kliem, Anna Lohmann, Sebastian Fischer, Dirk Baier, Vera Clemens, Cedric Sachser, Hanna Kampling, Elmar Brähler, Jörg M. Fegert

**Affiliations:** ^1^Department of Social Welfare, Ernst-Abbe University of Applied Sciences Jena, Jena, Germany; ^2^Institute of Delinquency and Crime Prevention, Zurich University of Applied Sciences, Zurich, Switzerland; ^3^University of Zurich, Zürich, Switzerland; ^4^Clinic for Child and Adolescent Psychiatry/Psychotherapy, Ulm University, Ulm, Germany; ^5^Department of Psychosomatic Medicine and Psychotherapy, Justus Liebig University Giessen, Giessen, Germany; ^6^Department of Psychosomatic Medicine and Psychotherapy, University Medical Center of the Johannes Gutenberg University Mainz, Mainz, Germany; ^7^Department of Medical Psychology and Sociology, Leipzig University, Leipzig, Germany

**Keywords:** well-being, self-report questionnaire, population norms, psychometrics, measurement invariance

## Abstract

**Introduction:**

The World Health Organization-Five Well-Being Index (WHO-5) is a widely used self-report measure for evaluating mental well-being in both general and clinical populations. This study examines the psychometric properties of the WHO-5 using a large, representative sample of the adult population in Germany (*N* = 2,515) and presents updated population norms.

**Methods:**

Analyses included item-level statistics such as means, standard deviations, and inter-item correlations. Construct validity was evaluated through correlations with measures of depression (PHQ-2), anxiety (GAD-2), somatic symptoms (SSS-8), and loneliness (UCLA Loneliness Scale). Internal consistency was measured using coefficient omega, while factorial validity was tested through confirmatory factor analysis based on a one-factor model. Measurement invariance was assessed across gender and age groups using multi-group confirmatory factor analyses. Population norms are reported for the total sample and various age groups.

**Results:**

The findings confirm the strong psychometric properties of the WHO-5, including its internal consistency and construct validity. Measurement invariance results support comparability of scores across gender and age. The updated norms offer.

**Discussion:**

These updated norms support the continued implementation of the WHO-5 as a practical tool for population-based prevention and mental health care planning.

## 1 Introduction

The World Health Organization-Five Well-Being Index (WHO-5) is an internationally recognized instrument for assessing subjective psychological well-being. Initially introduced in the late 1990s, the WHO-5 has gained widespread use in both research and clinical practice due to its brevity and generic nature, which facilitate its application across diverse populations (see Topp et al., 2015). The WHO-5 was derived from the longer WHO-10, itself an abridged version of a 28-item instrument developed during a multicenter European study (Topp et al., 2015). Due to its generic structure, the WHO-5 is suitable for comparisons between general population norms and clinical populations, irrespective of specific diagnoses. It is thus widely used as a benchmark for monitoring remission or assessing well-being in various contexts [e.g., older adults (Allgaier et al., 2013), individuals with diabetes (George and Randhawa, 2024; Sommer et al., 2024), stroke survivors (Damsbo et al., 2020), cancer patients (Aerts et al., 2014), individuals with alcohol use disorders (Elholm et al., 2011), individuals with schizophrenia (Fekih-Romdhane et al., 2024), individuals with sleep disturbances (Hartwig et al., 2019), individuals with multiple sclerosis (Andreasen et al., 2010), individuals with personality disorders (Lara-Cabrera et al., 2020), and individuals with bereavement (Reitsma et al., 2024); see Domenech et al., 2025 for an overview]. During the COVID-19 pandemic, the WHO-5 was widely used in cross-national studies (e.g., Gallemit et al., 2024; Lara-Cabrera et al., 2022), and even in settings affected by geopolitical crises (e.g., Alnaser et al., 2025). Validation studies in various languages, including Chinese, German, Malay, Swedish, Turkish, Azerbaijani, and Arabic, have consistently shown high internal consistencies (e.g., Aliyev et al., 2024; Brähler et al., 2007; Kassab Alshayea, 2023; Khosravi et al., 2015; Eser et al., 2019; Faruk et al., 2021; Fung et al., 2022; Löve et al., 2014; Suhaimi et al., 2022) ranging from α = 0.75 (Bangla version; Faruk et al., 2021) to α = 0.94 (Persian sample; Khosravi et al., 2015). Accordingly, confirmatory factor analyses (CFA) conducted across diverse populations consistently support the WHO-5’s unidimensional structure (e.g., Guðmundsdóttir et al., 2014; Perera et al., 2020; Cosma et al., 2022; Sischka et al., 2025). Measurement invariance across cultures has been extensively tested, albeit with mixed findings (e.g., Sischka et al., 2020; Caycho-Rodríguez et al., 2023; Jami and Kemmelmeier, 2020). Some studies demonstrated invariance across gender (e.g., Fekih-Romdhane et al., 2023; Perera et al., 2020; Yang et al., 2023) and age groups (e.g., Yang et al., 2023; Cosma et al., 2022). However, systematic investigations of measurement invariance across clinical and non-clinical samples remain scarce (e.g., Cosma et al., 2022; Eser et al., 2019), highlighting the need for further research in this area. The construct validity of the WHO-5 has been robustly supported through substantial correlations with a wide range of conceptually related constructs. Strong negative associations have been documented with depressive symptoms (e.g., de Wit et al., 2007; Gallemit et al., 2024; Möller-Leimkühler et al., 2007), generalized anxiety (e.g., Gallemit et al., 2024; Pheko et al., 2023; Fekih-Romdhane et al., 2024), somatic complaints (e.g., Brähler et al., 2007; Cosma et al., 2022; Sischka et al., 2025), and loneliness (e.g., Sischka et al., 2025; Pheko et al., 2023). Positive correlations with quality of life, overall life satisfaction, and other measures of well-being (e.g., Clarke et al., 2011; Lambert et al., 2014) further support the construct validity of the WHO-5. Although the WHO-5 has been extensively validated, studies involving representative samples of the general population remain limited. Existing German norms, based on data collected in 2004, are now outdated (Brähler et al., 2007). Profound societal and health-related changes have created a pressing need for updated benchmarks for the general population. Recent normative data from countries like Denmark (Moeller et al., 2023) highlight the importance of deriving updated reference values from large, representative community samples. Accordignly, this study aims to address this gap by evaluating the psychometric properties of the WHO-5 and providing updated norms based on a representative sample of the German population.

## 2 Methods and materials

### 2.1 Procedure

The WHO-5 was administered as part of a comprehensive survey conducted by Leipzig University between June and October 2021. Data collection was managed by USUMA Markt- und Sozialforschung, an independent research institute specializing in social and market research (e.g., Kliem et al., 2015; Kliem et al., 2016). The survey had three main objectives: (1) to estimate the prevalence of physical and mental health conditions and associated risk behaviors, (2) to explore contributing factors to these conditions, and (3) to validate psychological instruments and update German population norms.

The survey included two components. First, interviewers gathered demographic and household information through structured interviews aligned with the standards of the German Federal Statistical Office (Statistisches Bundesamt). Second, participants independently completed paper-based questionnaires in the interviewer’s presence but without their direct involvement. Interviewers remained available to address any questions. All participants provided informed consent, and those under 18 years required parental consent. Confidentiality and data protection measures were communicated in detail to all participants. The study adhered to the Declaration of Helsinki (World Medical Association, 2024) and was approved by the Ethics Committee of the Medical Faculty at Leipzig University (AZ: 298/21-ek).

### 2.2 Sample description

**Figure 1:**
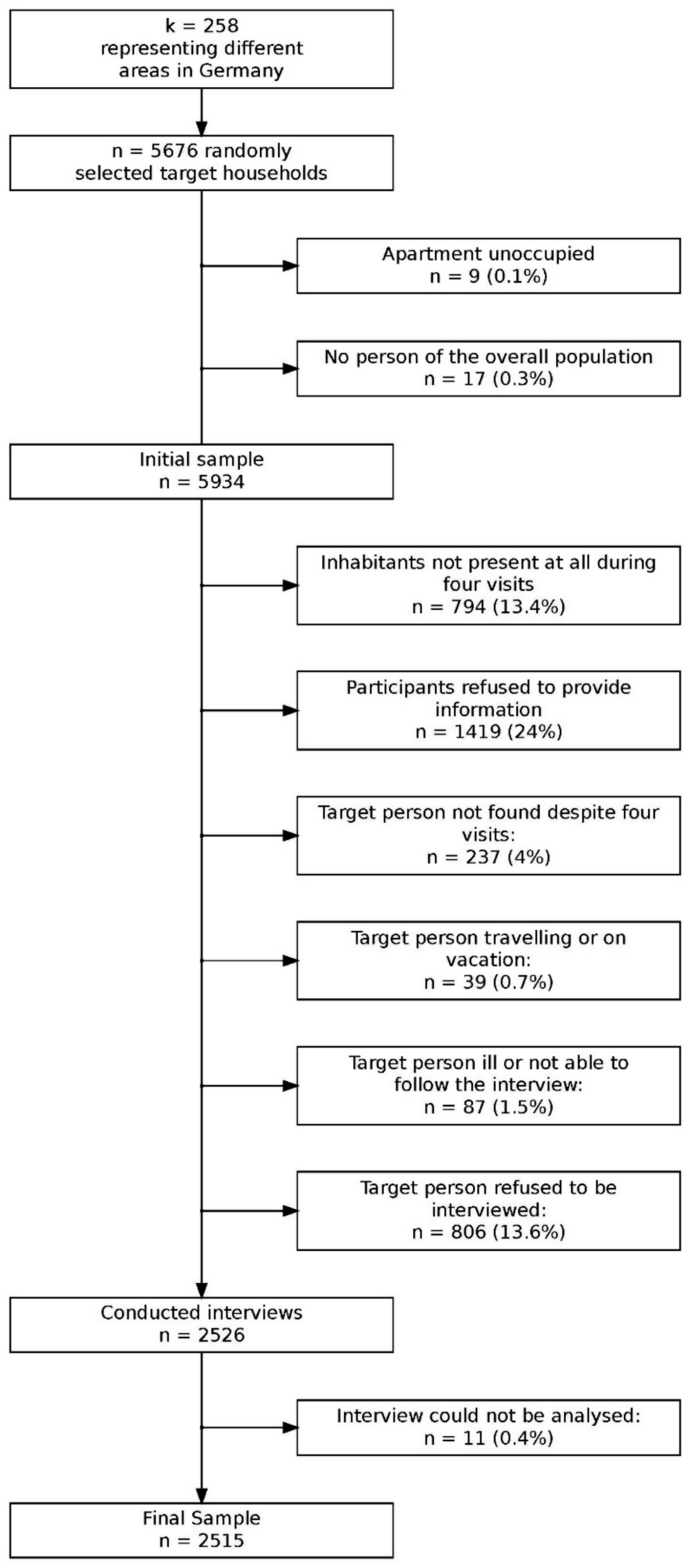
Flowchart of sampling procedure and reasons for nonparticipation.

The survey utilized the ADM (Arbeitskreis Deutscher Markt- und Sozialforschungsinstitute) sampling system to generate a representative sample of the German general population. Sampling involved three stages: (1) regional stratification to identify 258 sampling points across Germany, (2) random selection of 5,676 households using a random-route procedure, and (3) selection of target individuals within households using a Kish grid (Kish, 1949). Of the *K* = 5,908 households contacted, *N* = 2,515 completed the survey, resulting in a response rate of 42.6%. Data from *N* = 2,515 participants (51.6% female) were analyzed. Detailed sample characteristics are provided in Table 1, and an overview of the sampling procedure is shown in Figure 1. Additional descriptive statistics for the sample, stratified by the same groups used in the measurement invariance analyses and by normative comparison groups (see below), are available in the Supplemental material (see Supplementary Tables S1–S3).

**Table 1:** Demographic characteristics of the study sample.

	Male	Female	Diverse	Total
	(*N* = 1,217)	(*N* = 1,297)	(*N* = 1)	(*N* = 2,515)
Age				
*M* (*SD*)	49.5 (18.2)	50.6 (17.9)	80.0 (−)	50.1 (18.0)
Median [min, max]	51.0 [16.0, 101]	51.0 [16.0, 93.0]	80.0 [80.0, 80.0]	51.0 [16.0, 101]
Age categories				
16–24	124 (10.2%)	101 (7.8%)	0 (0%)	225 (8.9%)
25–34	199 (16.4%)	190 (14.6%)	0 (0%)	389 (15.5%)
35–44	148 (12.2%)	219 (16.9%)	0 (0%)	367 (14.6%)
45–54	204 (16.8%)	229 (17.7%)	0 (0%)	433 (17.2%)
55–64	266 (21.9%)	221 (17.0%)	0 (0%)	487 (19.4%)
65–74	177 (14.5%)	199 (15.3%)	0 (0%)	376 (15.0%)
75+	99 (8.1%)	138 (10.6%)	1 (100%)	238 (9.5%)
Nationality				
German	1,166 (95.8%)	1,243 (95.8%)	1 (100%)	2,410 (95.8%)
Not German	51 (4.2%)	53 (4.1%)	0 (0%)	104 (4.1%)
Missing	0 (0%)	1 (0.1%)	0 (0%)	1 (0.0%)
Marital status				
Married/living together	511 (42.0%)	490 (37.8%)	0 (0%)	1,001 (39.8%)
Married/separated	24 (2.0%)	20 (1.5%)	0 (0%)	44 (1.7%)
Single	448 (36.8%)	349 (26.9%)	0 (0%)	797 (31.7%)
Divorced	170 (14.0%)	234 (18.0%)	0 (0%)	404 (16.1%)
Widowed	62 (5.1%)	202 (15.6%)	1 (100%)	265 (10.5%)
Missing	2 (0.2%)	2 (0.2%)	0 (0%)	4 (0.2%)

M, mean values; SD, standard deviation.

### 2.3 Measures

This survey was designed to serve various epidemiological research purposes; however, only the measures relevant to the validation process are discussed in this paper. Alongside comprehensive demographic data (see Table 1), information on health-related behaviors, including the number of sick days, doctor visits, and hospital admissions, was collected. The following instruments were utilized to validate the scales examined in this study.

#### 2.3.1 The World Health Organization-five well-being index

The World Health Organization-Five Well-Being Index (WHO-5) (Bech et al., 2003; German version: Brähler et al., 2007) consists of five positively phrased items assessing well-being over the past 14 days. Participants rate each statement on a scale from 0 (at no time) to 5 (all of the time), yielding a total score range of 0–25. Scores are conventionally converted to a percentage scale ranging from 0 (lowest well-being) to 100 (highest well-being) by multiplying the raw score by four (Bech et al., 2003).

#### 2.3.2 The patient health questionnaire-4

The patient health questionnaire-4 (PHQ-4) (Löwe et al., 2010; Wicke et al., 2022) assess symptoms of depression (PHQ-2) and generalized anxiety disorder (GAD-2) on subscales of two items each. Each item is rated on a four-point scale (0 = not at all to 3 = nearly every day), resulting in subscale scores ranging from 0 to 6. Higher scores indicate greater symptom severity. Internal consistency for the PHQ-2, and GAD-2 in this study was acceptable to good (PHQ-2: α = 0.82, *ω* = 0.82; GAD-2: α = 0.76, ω = 0.77, respectively).

#### 2.3.3 UCLA loneliness scale (short form)

Loneliness was measured using a three-item version of the UCLA Loneliness Scale (Hughes et al., 2004; German version: Klein et al., 2021). Participants rated items on a five-point Likert scale (0 = never to 4 = very often), with higher scores indicating greater loneliness. The total score ranges from 0 to 12. The German version demonstrated very good reliability in this study (α = 0.90, ω = 0.91).

#### 2.3.4 Somatic symptom scale

The Somatic symptom scale (SSS-8) (Gierk et al., 2014) measures somatic symptom burden based on eight items, each rated on a five-point scale (0 = not at all to 4 = very strongly). Participants reported how much they had been affected by specific complaints over the past 7 days, with higher scores indicating higher burden. Internal consistency in the present sample was α = 0.86, ω = 0.90.

#### 2.3.5 European health interview survey quality of life 8-item index

The European health interview survey quality of life 8-item index (EUROHIS-QOL) (Schmidt et al., 2006; German version: Brähler et al., 2007; Hettich et al., 2022) is a quality of life measure consisting of eight items (overall QoL, general health, energy, daily living activity, self-esteem, social relationships, finances, and home). However, conceptually the psychological, physical, social and environmental domains are each represented by two items. All answer scales have a 5-point response format on a Likert scale, ranging from “not at all” to “completely.” The overall QOL score is formed by a simple summation of scores on the eight items, with higher scores indicating better QOL. Each item is answered individually on a five-point scale. Internal consistency in the sample at hand was α = 0.90, ω = 0.96.

### 2.4 Statistical analysis

#### 2.4.1 Missing data

The proportion of missing responses for the WHO-5 items ranged from 0.08 to 0.20%. To address missing data, multiple imputation via chained equations was applied, following the procedure described in van Buuren and Groothuis-Oudshoorn (2011). Sociodemographic variables and all scales relevant for assessing construct validity were included in the imputation model to predict missing values. Predictive mean matching was utilized to ensure plausible item values by selecting observed values closest to the predicted values (ŷ). The imputations were carried out using the “mice” package in R (van Buuren and Groothuis-Oudshoorn, 2011). All subsequent analyses were conducted on one imputed dataset.

#### 2.4.2 Item characteristics

Means (*M*), standard deviations (*SD*), kurtosis (*Kurt*), and skewness (*Skew*) for all WHO-5 items were calculated for the entire sample and for subgroups based on gender. Additionally, inter-item correlations (*r*) were calculated providing insight into the degree of association between items, indicating whether items measure similar aspects of the underlying construct. Furthermore, item-test correlations (*r_it_*) were calculated to assess the extent to which each item correlates with the sum of the remaining items. For this purpose, the item under consideration was excluded from the total score to avoid artificial inflation of the correlation coefficient and to obtain a corrected item-total correlation. Cronbach’s alpha if one item deleted (α^−1^) was calculated to examine the potential impact of each item on overall scale reliability; decreases in alpha following item deletion indicate that the item contributes positively to internal consistency, whereas increases suggest that the item may not align well with the scale’s overall structure. Lastly, we calculated item (*P_i_*) and total score difficulty (*P_t_*) by transforming raw scores to a standardized scale ranging from 0 to 100. This linear transformation was based on the minimum and maximum possible scores per item, allowing for comparability across items. Group differences in item means were assessed using Cohen’s d to quantify effect sizes.

#### 2.4.3 Construct validity

Construct validity was assessed by examining correlations between the WHO-5 and scales measuring depression (PHQ-2), anxiety (GAD-2), loneliness (UCLA Loneliness Scale-3), somatization (SSS-8), and quality of life (EROHIS-QOL-8). The following hypotheses guided the analysis: higher well-being would be associated with (a) lower scores on depression, (b) lower scores on anxiety, and (c) lower scores on somatization. Additionally, it was hypothesized that well-being would positively correlate with quality of life. In addition to the correlation analyses, we estimated a structural equation model (SEM) to examine the construct validity of the WHO-5 using latent variables. The model included the WHO-5 as a latent factor and its associations with conceptually related constructs.

#### 2.4.4 Internal consistency

To provide a robust measure of internal consistency, McDonald’s *ω* was calculated using the “semTools” package in R (Jorgensen et al., 2021). This approach complements the use of Cronbach’s α, which can be sensitive to certain assumptions (McNeish, 2018).

#### 2.4.5 Factorial validity and measurement invariance

To confirm the unidimensional structure of the WHO-5, confirmatory factor analyses (CFA) were conducted using the “lavaan” package in R (Rosseel, 2019). The analyses employed weighted least square means and variance adjusted (WLSMV) estimation, which is suitable for categorical data. To evaluate goodness-of-fit of the relevant model, the following four criteria were considered. While the RMSEA and the 90% confidence interval both assess absolute model fit, the two additionally calculated criteria (Comparative Fit Index [CFI] and Tucker Lewis Index [TLI]) measure relative model fit compared to the “null” model. RMSEA values < 0.050 represent a close fit, values between 0.050 and 0.080 represent a reasonably close fit, and values > 0.080 represent an unacceptable model (Hu and Bentler, 1999). Regarding CFI and TLI, Hu and Bentler (1999) suggested a CFI and TLI > 0.900 for an adequate fit and a CFI and TLI > 0.950 for a good model fit. In addition, the Standardized Root Mean Square Residual (SRMR) was considered as an indicator of the average standardized residuals between observed and predicted covariances; SRMR values < 0.080 are generally interpreted as indicative of good fit (Hu and Bentler, 1999). Measurement invariance (MI) was assessed using multiple group confirmatory factor analysis (MGCFA), adhering to the procedures recommended by Wu and Estabrook (2016). The theta parameterization was applied, with models identified by fixing latent factor means and variances to 0 and 1, setting item intercepts to 0, and constraining residual variances to 1.

Five nested models were tested: (i) configural invariance (unconstrained except for identification constraints), (ii) threshold invariance (equal thresholds across groups), (iii) weak invariance (equal factor loadings), (iv) strong invariance (equal intercepts), and (v) full invariance (equal residual variances). The parameter constraints for each model are visualized in Supplementary Figure S1 and described in detail in Supplementary Table S4. Cut-off criteria by Chen (2007) were applied, with CFI changes of < −0.01 and RMSEA changes of ≥0.015 signaling lack of invariance. MGCFA analyses were conducted across gender, age (below and above median age), and combined age-gender groups. Non-binary cases were excluded from the gender-based analyses due to their small numbers. In addition to age and sex, we also examined measurement invariance across subgroups defined by anxiety symptom severity (GAD-2), depression symptom severity (Löwe et al., 2004) and somatic symptom burden (SSS-8), as differential response behavior in these groups may affect comparability of scores. For subgroup classification, we applied established cut-off scores [GAD-2: ≥3.0 (Löwe et al., 2010); PHQ-2 (Löwe et al., 2010); ≥3.0, and SSS-8 (Gierk et al., 2014): ≥8.0] for each scale to distinguish between individuals with clinically relevant versus non-clinically relevant symptom levels.

## 3 Results

### 3.1 Item characteristics

Supplementary Table S5 provides a detailed overview of totel score and item-level statistics. The WHO-5 yielded a mean score of *M* = 16.89 (*SD* = 5.74), with a negatively skewed distribution (*Skew* = −0.90) and near-normal kurtosis (*Kurt* = 0.08). Difficulty for the total score was *P_t_* = 67.56, indicating a generally positive self-reported well-being in the sample. On item level mean scores ranged from *M* = 3.36 (#4) to 3.55 (#2), with moderate variability (*SD* = 1.14 to 1.32), indicating generally high levels of well-being within the sample. All items showed negative skewness (*Skew* = −1.06 to −0.75), suggesting a response pattern skewed toward higher endorsements. Item difficulty ranged between *P_i_* = 67.25 (#4) and *p* = 71.08 (#2), reflecting moderate item difficulty. Corrected item-total correlations (*r_it_*) were consistently high (*r^it^ = 0*.83 to 0.87), indicating strong discrimination and conceptual alignment of the items with the overall construct. Cronbach’s alpha remained stable across all deletion scenarios (α^−1^ = 0.93 to 0.94), demonstrating that each item contributes positively to the internal consistency of the scale. Supplementary Table S6 provides a detailed overview of the inter-item correlations for the WHO-5 ranging from *r* = 0.84 (95% CI [0.83, 0.85]) to *r* = 0.76 (95% CI [0.74, 0.77]). Furthermore, Supplementary Table S7 reports *M* and *SD* for gender subgroups. Male participants generally reported higher mean well-being scores and exhibited lower variability on most items. Effect sizes for gender differences were small, with Cohen’s d ranging from ES = 0.05 (#1; 95% CI [0.03, 0.13]) to 0.13 (#4; 95% CI [−0.05, 0.20]).

### 3.2 Construct validity

Correlations between the WHO-5 and related scales supported its construct validity. As hypothesized, the WHO-5 showed strong negative correlations with PHQ-2 [*r*(2,413) = −0.64, *p* < 0.001], GAD-2 [*r*(2,413) = −0.53, *p* < 0.001], UCLA Loneliness Scale-3 [*r*(2,413) = −0.54, *p* < 0.001], and SSS-8 [*r*(2,413) = −0.59, *p* < 0.001]. A strong positive correlation was observed with EROHIS-QOL-8 [*r*(2,413) = 0.68, *p* < 0.001]. A comprehensive correlation table is provided in Supplementary Table S8. In Supplementary Table S9, the correlations between individual WHO-5 items and theoretically relevant constructs are reported, providing evidence for construct-related validity on item level. The SEM results showed the expected pattern of correlations, with strong negative associations with depression and anxiety, and moderate associations with the other health indicators. Detailed parameter estimates are reported in Supplementary Table S10.

### 3.3 Population norms

Percentile ranks of WHO-5 scores for the overall sample are presented in Table 2. Additional norms stratified by gender and age are available in Supplementary Tables S11, S12.

**Table 2:** Population based norms (cumulative percentiles) of the WHO-5 scores (total sample).

WHO-5	Total	Age 16–24	Age 25–34	Age 35–44	Age 45–54	Age 55–64	Age 65–74	Age 75+
0	0.3 [0.12, 0.52]	<0.1	<0.1	0.3 [0, 0.8]	0.2 [0, 0.7]	0.6 [0, 1.4]	<0.1	0.8 [0, 2.1]
4	0.7 [0.36, 1.03]	<0.1	<0.1	1.1 [0.3, 2.2]	0.5 [0, 1.2]	0.8 [0.2, 1.6]	0.3 [0, 0.8]	2.5 [0.8, 4.61]
8	1.4 [0.91, 1.83]	<0.1	0.5 [0, 1.3]	1.6 [0.5, 3]	0.5 [0, 1.2]	1.6 [0.6, 2.9]	1.3 [0.3, 2.7]	4.6 [2.1, 7.6]
12	2.7 [2.07, 3.42]	1.8 [0.4, 3.6]	1.5 [0.5, 2.8]	1.9 [0.5, 3.5]	2.1 [0.9, 3.5]	2.5 [1, 3.9]	2.7 [1.1, 4.3]	8.4 [5, 12.2]
16	3.9 [3.14, 4.61]	1.8 [0.4, 3.6]	2.8 [1.3, 4.4]	2.7 [1.1, 4.6]	3.2 [1.6, 4.8]	3.5 [1.8, 5.1]	4.3 [2.4, 6.4]	10.5 [6.7, 14.7]
20	6.8 [5.84, 7.87]	3.6 [1.3, 6.2]	4.4 [2.3, 6.4]	6.3 [4.1, 8.7]	5.3 [3.5, 7.6]	5.7 [3.9, 7.8]	9.3 [6.6, 12.2]	16 [11.3, 20.6]
24	8 [6.88, 9.11]	3.6 [1.3, 6.2]	5.1 [3.1, 7.5]	7.4 [4.9, 10.1]	6.7 [4.6, 9.2]	7 [4.9, 9.2]	10.1 [7.2, 13]	18.9 [13.9, 23.9]
28	9.5 [8.35, 10.74]	4 [1.8, 6.71]	6.2 [3.9, 8.7]	7.9 [5.4, 10.6]	7.6 [5.3, 10.2]	10.5 [7.8, 13.1]	10.4 [7.4, 13.3]	22.7 [17.6, 28.2]
32	11.3 [10.02, 12.68]	5.8 [3.1, 8.9]	7.7 [5.1, 10.3]	9.5 [6.5, 12.5]	9 [6.7, 11.8]	11.3 [8.4, 14]	10.4 [9.3, 15.7]	27.7 [22.3, 33.6]
36	12.9 [11.53, 14.35]	6.7 [3.6, 9.8]	8.5 [5.9, 11.3]	10.9 [7.6, 14.2]	9.5 [6.9, 12.2]	12.7 [9.9, 15.6]	16 [12.5, 19.7]	31.1 [25.2, 37]
40	15.7 [14.27, 17.18]	9.3 [5.8, 13.3]	9.8 [7.2, 12.6]	12.5 [9.5, 15.8]	12 [9.2, 15.2]	15.6 [12.3, 18.7]	20.2 [16.2, 24.5]	36.1 [30.3, 42.4]
44	18.4 [16.94, 19.92]	12 [8, 16.4]	11.3 [8.5, 14.4]	14.7 [11.4, 18.3]	14.8 [11.5, 18.2]	18.7 [15.2, 22]	23.1 [18.9, 27.7]	40.3 [34.5, 46.6]
48	21.3 [19.72, 22.9]	15.6 [11.1, 20.4]	14.1 [11.1, 17.7]	18 [14.4, 21.81]	17.3 [13.9, 20.8]	20.7 [17.2, 24.2]	27.4 [23.4, 31.9]	42.4 [36.1, 48.71]
52	24 [22.39, 25.65]	17.3 [12.4, 22.2]	15.4 [12.1, 19]	21.5 [17.4, 25.6]	19.6 [15.9, 23.1]	23.4 [19.7, 27.1]	29.3 [25.29, 34]	48.7 [42.4, 55]
56	27.2 [25.49, 28.91]	22.2 [16.4, 27.6]	17.5 [13.9, 21.3]	24 [19.9, 28.1]	24 [20.1, 27.7]	26.7 [22.6, 30.4]	31.4 [27.09, 36.2]	52.5 [46.59, 58.4]
60	32.4 [30.66, 34.23]	28 [21.8, 33.8]	20.3 [16.5, 24.4]	28.6 [24, 33.2]	29.8 [25.4, 33.7]	32.4 [28.1, 36.6]	37 [31.9, 41.8]	59.7 [53.4, 66]
64	36 [34.16, 37.85]	30.2 [24, 36.01]	22.9 [18.8, 27]	33 [28.1, 37.6]	32.6 [27.9, 36.7]	36.6 [32.2, 40.7]	41 [36.2, 46]	64.7 [59.2, 70.6]
68	42.4 [40.52, 44.25]	34.2 [28, 40.9]	28.5 [23.9, 33.2]	38.4 [33.5, 43.3]	39.3 [34.6, 43.9]	44.4 [39.99, 48.9]	48.4 [43.59, 53.5]	71.4 [66, 76.51]
72	49.5 [47.47, 51.33]	40.4 [33.8, 47.1]	33.9 [29.3, 38.6]	46.3 [41.4, 51.5]	46 [41.1, 50.1]	53 [48.5, 57.5]	56.9 [52.1, 62]	75.6 [70.2, 80.71]
76	57.5 [55.47, 59.4]	47.1 [40.4, 53.81]	41.6 [36.5, 46.5]	52.3 [47.4, 58]	56.1 [51, 60.71]	63.2 [58.89, 67.6]	56.9 [61.2, 70.2]	78.6 [73.09, 83.6]
80	77.7 [76.02, 79.4]	63.1 [56.4, 70.2]	68.1 [63.2, 72.5]	76.3 [71.9, 80.91]	79.7 [75.8, 83.4]	63.2 [78.6, 85.2]	81.6 [77.7, 85.4]	91.2 [87.8, 94.5]
84	82.1 [80.56, 83.66]	71.6 [66.19, 77.8]	73.5 [68.9, 77.6]	80.9 [77.09, 85.3]	83.1 [79.7, 86.6]	86.4 [83.4, 89.3]	85.6 [81.9, 89.4]	91.6 [88.2, 95]
88	86.9 [85.49, 88.19]	81.8 [76.9, 86.71]	79.2 [75.1, 83.3]	84.7 [81.2, 88.6]	87.5 [84.3, 90.5]	90.8 [88.1, 93.2]	89.4 [85.9, 92.6]	94.5 [91.6, 97.1]
92	90.5 [89.34, 91.77]	86.2 [81.8, 90.7]	86.6 [83, 90]	88.6 [85.3, 91.8]	91.5 [88.9, 94]	93.4 [91, 95.5]	91.5 [88.6, 94.4]	95 [92, 97.5]
96	92 [90.97, 93.2]	87.1 [82.7, 91.6]	87.4 [84.1, 90.7]	90.5 [87.2, 93.5]	93.3 [91, 95.6]	95.1 [93, 96.9]	93.6 [91.2, 96]	95.8 [93.3, 97.9]
100	>99.9	>99.9	>99.9	>99.9	>99.9	>99.9	>99.9	>99.9

All raw scores were multiplied by 4 in order to transform the original WHO-5 score range from 0 to 25 to a standardized scale of 0–100, as recommended in the scoring guidelines; Values in square brackets indicate the 95% confidence interval based on 1,000 bootstrap samples.

### 3.4 Internal consistency

The internal consistency of the WHO-5 was excellent, with both Cronbach’s α and McDonald’s *ω* yielding values of 0.95 for the full sample.

### 3.5 Factorial validity

CFA results provided evidence of unidimensionality for the WHO-5. Fit indices indicated good model fit with robust CFI = 0.977, robust TLI = 0.954, and SRMR = 0.016. Despite the elevated RMSEA value, the overall fit indices, including the robust CFI, TLI, and SRMR, provide compelling evidence supporting a well-fitting unidimensional model of the WHO-5. The robust RMSEA was calculated at 0.17 (90% CI [0.153, 0.186]), which may be attributed to its sensitivity to model simplicity (see discussion). Considering the strong fit indicated by the other indices, this discrepancy is unlikely to compromise the validity of the model. Standardized factor loadings ranged from 0.89 to 0.94, reinforcing the scale’s unidimensional structure (see Supplementary Table S5). Path diagrams are included in Supplementary Figures S2, S3.

### 3.6 Measurement invariance

Measurement invariance analyses yielded satisfactory fit indices across all steps and groups. The fit statistics are presented in Supplementary Table S13, supporting the comparability of WHO-5 scores across gender and age groups. With regard to anxiety, depression, and somatic symptom severity, the analyses indicated largely consistent model fit across configural, threshold, metric, and scalar levels. These findings support the robustness of the WHO-5 across varying levels of psychological and somatic symptomatology and are presented in Supplementary Table S14.

## 4 Discussion

This study evaluated the psychometric properties of the WHO-5 in a large, representative sample of the German general population. The results demonstrated high internal consistency, as evidenced by McDonald’s ω. Despite the high RMSEA, the overall fit indices (robust CFI, TLI, SRMR) provide strong evidence for a well-fitting unidimensional model of the WHO-5. It has been shown that RMSEA has significant issues with simpler models that have few degrees of freedom. This is particularly relevant for simple path models and CFAs, which often have relatively few df. In such cases, RMSEA can incorrectly indicate poor model fit, even when the model fits the data well (Kenny et al., 2015). The problem arises from the construct of RMSEA as an absolute fit index that incorporates model complexity (Hu and Bentler, 1999). To account for simplicity, RMSEA applies a penalty for fewer df. This penalty can cause models with few df to exhibit poor RMSEA values, even when they fit the data well. Kenny et al. (2015) demonstrated through simulations that models with few df might even show poor RMSEA values despite a non-significant chi-square test (indicating no significant discrepancy between the model and the data). This conclusion is further supported by the finding that in the more constrained models of the MGCFA, which are characterized by a greater number of df, the RMSEA values consistently indicate an acceptable model fit. This suggests that the inclusion of additional constraints and the resulting increase in degrees of freedom may mitigate the sensitivity of RMSEA, allowing it to reflect a more accurate assessment of model fit under these conditions. Correspondingly, the measurement invariance analyses confirmed comparable factor structures across gender and age subgroups, supporting the use of WHO-5 for comparisons across these demographics. Furthermore, measurement invariance analyses demonstrated that the WHO-5 functioned equivalently across individuals with and without elevated anxiety, depression, and somatic symptoms. Full scalar and strict invariance were established for all three constructs (GAD-2, PHQ-2, and SSS-8), indicating that comparisons of latent well-being scores across symptom groups are psychometrically valid. Additionally, correlations with related constructs, including depression, anxiety, loneliness, somatization, and quality of life, provided strong evidence for construct validity. These findings align with prior normative studies—such as Moeller et al. (2023) and affirm the WHO-5’s utility as a reliable and valid measure of well-being in general population settings. Moeller et al. (2023) found a slightly lower mean well-being in a comparable sample of the Danish general population (*M*_percentage-scale_ = 63.9, SD_percentage-scale_ = 22.0 compared to *M*_percentage-scale_ = 67.56, *SD*_percentage-scale_ = 22.96 in the study at hand). Differences may seem unexpected, as Denmark is consistently ranked among the world’s “happiest” countries. A possible explanation for the higher well-being scores in the German sample compared to the Danish one lies in methodological and contextual differences between the studies. A key distinction is the timing of data collection. The Danish data were gathered at the onset of the COVID-19 pandemic, a period marked by high uncertainty, social isolation, and economic concerns, which likely had a negative impact on subjective well-being. In contrast, the German data were collected in June 2021, when the pandemic situation had significantly improved. By that time, infection rates had declined following a strict winter lockdown, widespread restrictions were lifted, and the vaccination campaign was well advanced, contributing to a greater sense of security in the population. Compared to norm data from the German general population collected in 2004 (Brähler et al., 2007), the present study found significantly higher levels of well-being across all subsamples. This increase in well-being among the German population is likely attributable to a combination of long-term societal improvements and greater awareness of mental health. Between 2004 and 2021, Germany has experienced economic stability and growth, characterized by low unemployment rates (decline in unemployment from 10.5% in 2004 to 5.7% in 2021), improved healthcare access (e.g., increased coverage for psychotherapy, and broader access to preventive healthcare services), and an expansion of social security systems (e.g., introduction of the statutory minimum wage in 2015, strengthening of parental benefits through “Elterngeld” and “ElterngeldPlus”), all of which are key determinants of subjective well-being. Additionally, mental health awareness has increased, fostering greater acceptance of psychological support, improved access to therapy, and a broader adoption of self-care practices. These developments may have contributed to more effective coping mechanisms, enabling individuals to better manage stress and maintain higher well-being levels. Given these shifts, the findings underscore the importance of regularly updating well-being norms to reflect societal changes.

### 4.1 Limitations

While this study utilized data from a large representative sample, certain limitations should be acknowledged. The response rate, at 42.6%, is consistent with other general population studies (e.g., Kliem et al., 2024; Kliem et al., 2018) but raises concerns about potential non-response bias. Efforts were made to ensure representativeness. However, the lack of demographic information for non-responders limits the ability to assess the extent of such bias. Access to registry-based data, which requires governmental authorization in Germany, could mitigate this issue in future research. While the presented norms and psychometric properties can serve as valuable reference data for epidemiological and clinical research, the generalizability to clinical samples is limited. Nevertheless, population-based norms are essential for calculating metrics such as the Reliable Change Index (RCI; Jacobson and Truax, 1991) in clinical samples. Thus, despite being derived from a non-clinical population, these norms have direct clinical relevance by enabling the evaluation of meaningful individual-level changes in treatment contexts. Second, the study relies entirely on self-report data. Without external validation or objective behavioral or clinical measures, it cannot be ruled out that generalized negative self-evaluation might drive the observed association patterns (e.g., between well-being and depressive symptoms). Third, the data were collected exclusively within the German general population. While this ensures national representativeness, it may limit the generalizability of the findings to countries with different or more heterogeneous cultural norms and attitudes. However, given the extensive body of validation studies across diverse cultural contexts, it is reasonable to assume that the WHO-5 also functions reliably in other populations. Nevertheless, country-specific normative data are still needed, as cut-off values and score distributions may vary substantially. Moreover, it remains unclear how the COVID-19 pandemic has affected normative values in other countries, particularly in light of varying public health responses and demographic differences. Some countries implemented less restrictive infection control measures or were less severely impacted due to younger average population age structures - whereas Germany, as an aging society, may have experienced more pronounced effects on mental well-being. This underscores the importance of regularly updating national norms to account for such contextual and demographic variability. Fourth, the cross-sectional design of the study restricts conclusions regarding the predictive validity, test–retest reliability, and longitudinal measurement invariance of the German version of the WHO-5. To establish the temporal stability and predictive utility of the instrument, future research should adopt longitudinal approaches. Despite these limitations, the updated German WHO-5 norms provide valuable reference values for public mental health monitoring and screening purposes. Given its brevity, ease of administration, and strong psychometric properties, the WHO-5 is particularly well-suited for routine use in large-scale surveys, digital applications, and preventive healthcare. These updated norms therefore support the continued implementation of the WHO-5 as a practical tool for population-based prevention and mental health care planning.

## Data Availability

The datasets presented in this article are not readily available because the datasets generated and/or analyzed during the current study are not publicly available as ethics board approval did not include open data sharing. Questions concerning the data should be addressed to E. Brähler. Requests to access the datasets should be directed to Elmar.Braehler@medizin.uni-leipzig.de.
