# Supplementary material for: Psychometric evaluation and updated community norms of the WHO-5 well-being index, based on a representative German sample
Source: Front Psychol. 2025 Jul 29;16:1592614. doi: 10.3389/fpsyg.2025.1592614 (PMC12341540; doi:10.3389/fpsyg.2025.1592614)
Supplement: Supplementary file 1 [file Data_Sheet_1.pdf]

Supplementary Material

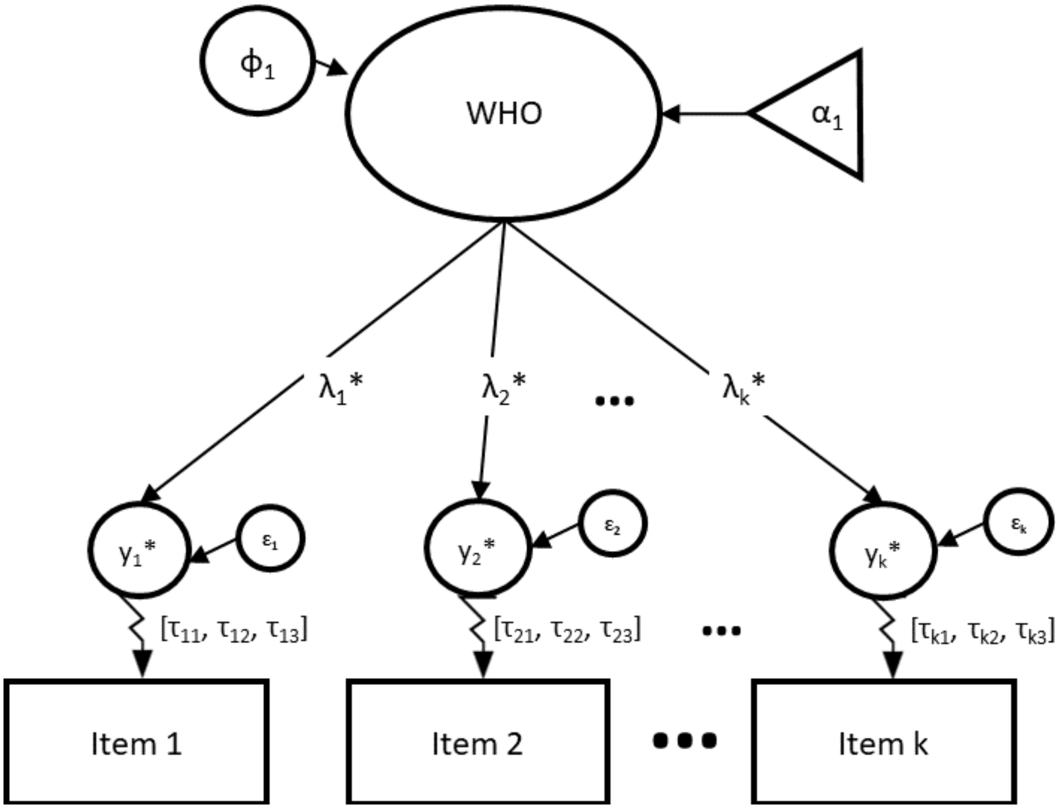

Supplementary Figure 1. MGCFA models for measurement invariance Analysis

(1) Configural invariance: Same CFA model fit in all groups;
(2) Threshold Invariance: thresholds $\tau 1_i ... \tau 4_i$ constrained to be equal across groups;
(3) Metric Invariance: additionally, factor loadings λ_i constrained to be equal across groups;
(4) Scalar invariance: additionally, intercepts constrained to be equal across groups;
(5) Residual invariance: additionally, residual variances ϵ_i constrained to be equal across groups.

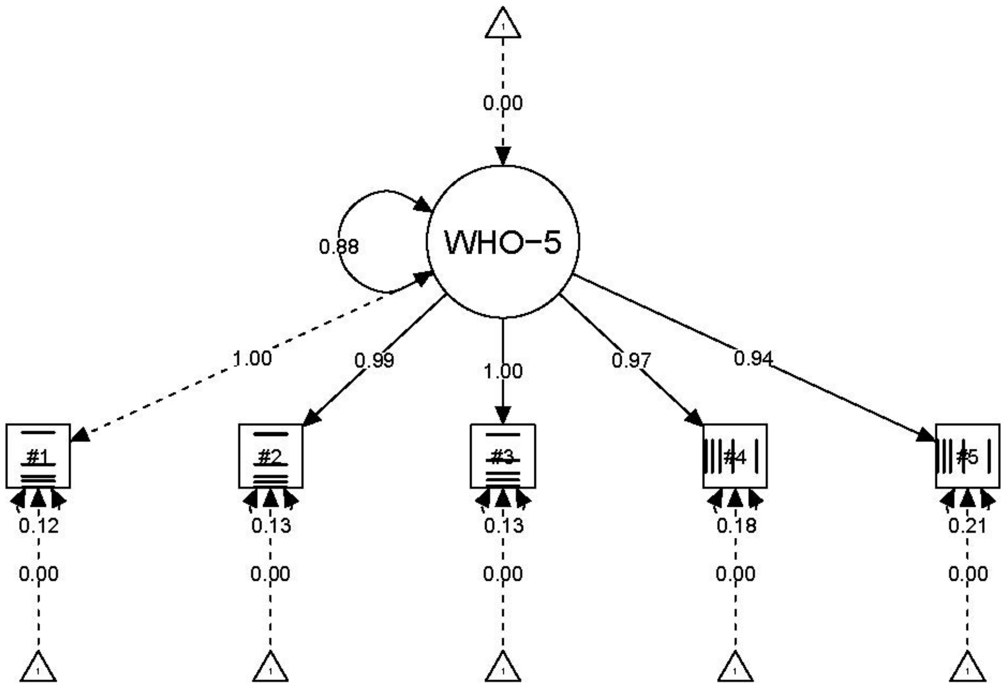

Supplementary Figure 2. One factor CFA model of the WHO-5

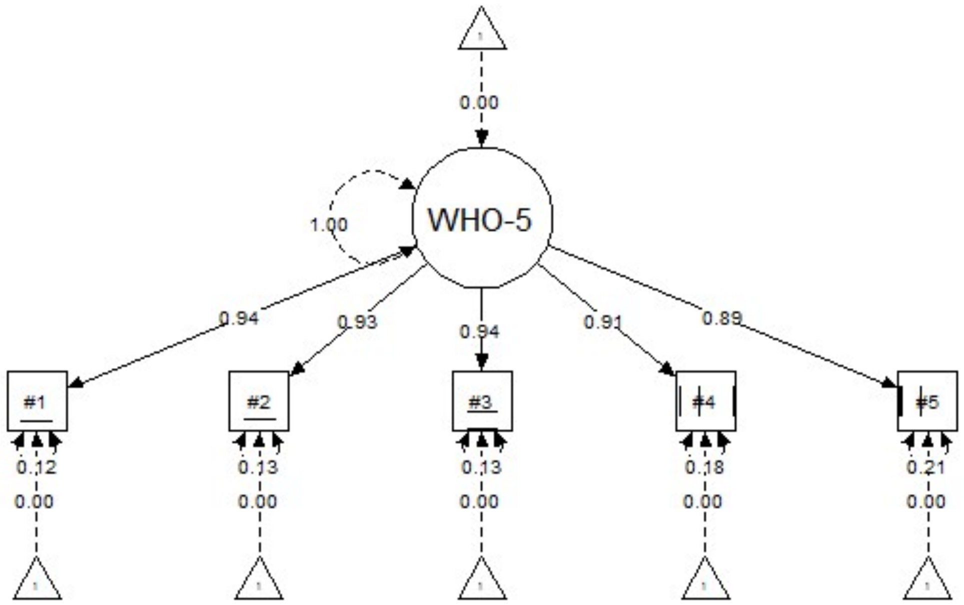

Supplementary Figure 3. One factor CFA model of the WHO-5 (standardized loading)

Supplementary Table 1. Demographic characteristics of the study sample by age and gender

	Male, Age < Median	Male, Age > Median	Female, Age < Median	Female, Age > Median	Total
	(*N*=615)	(*N*=649)	(*N*=602)	(*N*=648)	(*N*=2514)
Age					
M (*SD*)	34.4 (10.3)	35.5 (9.78)	65.0 (9.19)	65.7 (9.45)	50.1 (18.0)
Median [Min, Max]	34.0 [16.0, 51.0]	36.0 [16.0, 51.0]	63.0 [52.0, 101.0]	65.0 [52.0, 93.0]	51.0 [16.0, 101]
Nationality					
German	583 (94.8%)	603 (92.9%)	583 (96.8%)	640 (98.8%)	2409 (95.8%)
not German	32 (5.2%)	45 (6.9%)	19 (3.2%)	8 (1.2%)	104 (4.1%)
Missing	0 (0%)	1 (0.2%)	0 (0%)	0 (0%)	1 (0.0%)
Marital Status					
married/living together	201 (32.7%)	266 (41.0%)	310 (51.5%)	224 (34.6%)	1001 (39.8%)
married/separated	6 (1.0%)	7 (1.1%)	18 (3.0%)	13 (2.0%)	44 (1.8%)
single	357 (58.0%)	288 (44.4%)	91 (15.1%)	61 (9.4%)	797 (31.7%)
divorced	46 (7.5%)	81 (12.5%)	124 (20.6%)	153 (23.6%)	404 (16.1%)
widowed	4 (0.7%)	5 (0.8%)	58 (9.6%)	197 (30.4%)	264 (10.5%)
Missing	1 (0.2%)	2 (0.3%)	1 (0.2%)	0 (0%)	4 (0.2%)

Note. M = mean values; SD = standard deviation.

Supplementary Table 2. Demographic characteristics of the study sample by median age split

	Age <= Median	Age > Median	Total
	(*N*=1264)	(N=1251)	(*N*=2515)
Age			
M (*SD*)	35.0 (10.1)	65.4 (9.34)	50.1 (18.0)
Median [Min, Max]	35.0 [16.0, 51.0]	64.0 [52.0, 101]	51.0 [16.0, 101]
Gender			
Male	615 (48.7%)	602 (48.1%)	1217 (48.4%)
Female	649 (51.3%)	648 (51.8%)	1297 (51.6%)
Diverse	0 (0%)	1 (0.1%)	1 (0.0%)
Nationality			
German	1186 (93.8%)	1224 (97.8%)	2410 (95.8%)
not German	77 (6.1%)	27 (2.2%)	104 (4.1%)
Missing	1 (0.1%)	0 (0%)	1 (0.0%)
Marital Status			
married/living together	467 (36.9%)	534 (42.7%)	1001 (39.8%)
married/separated	13 (1.0%)	31 (2.5%)	44 (1.7%)
single	645 (51.0%)	152 (12.2%)	797 (31.7%)
divorced	127 (10.0%)	277 (22.1%)	404 (16.1%)
widowed	9 (0.7%)	256 (20.5%)	265 (10.5%)
Missing	3 (0.2%)	1 (0.1%)	4 (0.2%)

Note. *M* = mean values; *SD* = standard deviation.

Supplementary Table 3. Demographic characteristics of the study sample by age categories

	Age 16-24 (*N*=225)	Age 25-34 (*N*=389)	Age 35-44 (*N*=367)	Age 45-54 (*N*=433)	Age 55-64 (*N*=487)	Age 65-74 (*N*=376)	Age 75+ (*N*=238)	Total (*N*=2515)
Age								
M (*SD*)	20.1 (2.59)	29.5 (2.86)	39.6 (2.86)	49.9(2.86)	59.2 (2.79)	68.8 (2.66)	80.3 (4.09)	50.1 (18.0)
Median [Min, Max]	20.0 [16.0, 24.0]	29.0 [25.0,34.0]	40.0 [35.0, 44.0]	50.0 [45.0, 54.0]	59.0 [55.0, 64.0]	69.0 [65.0,74.0]	80.0 [75.0,101.0]	51.0 [16.0, 101.0]
Gender								
Male	124 (55.1%)	199 (51.2%)	148 (40.3%)	204 (47.1%)	266 (54.6%)	177 (47.1%)	99 (41.6%)	1217 (48.4%)
Female	101 (44.9%)	190 (48.8%)	219 (59.7%)	229 (52.9%)	221 (45.4%)	199 (52.9%)	138 (58.0%)	1297 (51.6%)
Diverse	0 (0%)	0 (0%)	0 (0%)	0 (0%)	0 (0%)	0 (0%)	1 (0.4%)	1 (0.0%)
Nationality								
German	216 (96.0%)	364 (93.6%)	339 (92.4%)	414 (95.6%)	472 (96.9%)	370 (98.4%)	235 (98.7%)	2410 (95.8%)
not German	9 (4.0%)	25 (6.4%)	28 (7.6%)	18 (4.2%)	15 (3.1%)	6 (1.6%)	3 (1.3%)	104 (4.1%)
Missing	0 (0%)	0 (0%)	0 (0%)	1 (0.2%)	0 (0%)	0 (0%)	0 (0%)	1 (0.0%)
Marital Status								
married/living together	7 (3.1%)	108 (27.8%)	192 (52.3%)	226 (52.5%)	220 (45.2%)	172 (45.7%)	76 (31.9%)	1001 (39.8%)
married/separated	0 (0%)	2 (0.5%)	7 (1.9%)	8 (1.8%)	16 (3.3%)	6 (1.6%)	5 (2.1%)	44 (1.7%)
single	217 (96.4%)	269 (69.2%)	113 (30.8%)	82 (18.9%)	75 (15.4%)	29 (7.7%)	12 (5.0%)	797 (31.7%)
divorced	0 (0%)	10 (2.6%)	51 (13.9%)	106 (24.5%)	135 (27.7%)	81 (21.5%)	21 (8.8%)	404 (16.1%)
widowed	0 (0%)	0 (0%)	2 (0.5%)	11 (2.5%)	40 (8.2%)	88 (23.4%)	124 (52.1%)	265 (10.5%)
Missing	1 (0.4%)	0 (0%)	2 (0.5%)	0 (0%)	1 (0.2%)	0 (0%)	0 (0%)	4 (0.2%)

Note. *M* = mean values; *SD* = standard deviation.

Supplementary Table 4. Parameter Constraints for MGCFA

Parameters	Threshold invariance	Metric Invariance	Scalar Invariance	Residual Invariance
Item loadings	free	equal across groups	equal across groups	equal across groups
Intercepts				
Items	constrained to 0 in first group	constrained to 0 in first group	equal (0) across groups	equal (0) across groups
Latent	constrained to 0 in all groups	constrained to 0 in all groups	constrained to zero in first group	constrained to zero in first group
Thresholds	equal across groups	equal across groups	equal across groups	equal across groups
Variances				
Residual	constrained to 1 in first group	constrained to 1 in first group	constrained to 1 in first group	equal (1) across groups
Latent	constrained to 1 in all groups	constrained to 1 in first group	constrained to 1 in first group	constrained to 1 in first group

Supplementary Table 5. Item statistics with descriptive and psychometric indices

Item	*M*	*SD*	*Skew*	*Kurt*	*P*	r_{it}	α^{-1}	λ
WHO-5	16.89	5.74	-0.90	0.08	67.56	-	-	-
cheerful and in good spirits (#1)	3.44	1.19	-1.06	0.53	69.30	0.86	0.93	0.94
calm and relaxed (#2)	3.49	1.18	-1.03	0.66	71.08	0.85	0.93	0.93
active and vigorous (#3)	3.34	1.30	-0.86	0.09	68.20	0.87	0.93	0.93
woke up feeling fresh and rested (#4)	3.27	1.35	-0.80	-0.09	67.25	0.84	0.93	0.90
daily life filled with things that interest me (#5)	3.35	1.29	-0.75	-0.19	67.90	0.83	0.94	0.88

Note. M = mean values; SD = standard deviation; Skew = skewness; Kurt = kurtosis; P = difficulty index (proportion of maximum possible score); r_{it} = item-total correlation; α^{-1} = Cronbach's alpha if the item is deleted; λ = standardized factor loading from confirmatory factor analysis (CFA).

Supplementary Table 6. Inter-item correlations with confidence intervals the WHO-5 items

Variable	1	2	3	4
1. #1				
2. #2	.84** [.83, .85]			
3. #3	.80** [.78, .81]	.80** [.79, .82]		
4. #4	.76** [.74, .77]	.78** [.76, .79]	.82** [.81, .84]	
5. #5	.78** [.77, .80]	.76** [.74, .77]	.79** [.78, .81]	.77** [.76, .79]

Note. Values in square brackets indicate the 95% confidence interval; * indicates $p < .05$, ** indicates $p < .001$.

Supplementary Table 7. Gender differences in the WHO-5 total score and individual item scores

	Male (N=1217)		Female (*N*=1297)		Group differences			
	M	*SD*	*M*	*SD*	*d* [95% CI]	*t*	*df*	*p*
WHO-5	17.19	5.63	16.62	5.83	0.1 [0.02,0.18]	2.49	2512	.013
cheerful and in good spirits (#1)	3.47	1.19	3.41	1.18	0.05 [-0.03,0.13]	1.16	2512	.246
calm and relaxed (#2)	3.55	1.14	3.43	1.20	0.1 [0.02,0.18]	2.72	2512	.007
active and vigorous (#3)	3.41	1.28	3.28	1.31	0.1 [0.02,0.18]	2.46	2512	.014
woke up feeling fresh and rested (#4)	3.36	1.32	3.19	1.37	0.13 [0.05,0.2]	3.28	2512	.001
daily life filled with things that interest me (#5)	3.40	1.28	3.31	1.30	0.07 [-0.01,0.15]	1.63	2512	.103

Note. *M* = mean values; *SD* = standard deviation; *d* = effect size Cohen's d; Values in square brackets indicate the 95% confidence interval.

Supplementary Table 8. Means (M), standard deviations (SD), and correlations with confidence intervals between the WHO-5 and scales measuring depression (PHQ-2), anxiety (GAD-2), loneliness (UCLA Loneliness Scale-3), somatization (SSS-8), and quality of life (EUROHIS-QOL-8)

Variable	M	SD	1	2	3	4	5
1. WHO-5	16.89	5.74					
2. PHQ-2	0.83	1.20	-.64** [-.66, -.62]				
3. GAD-2	0.78	1.12	-.53** [-.56, -.50]	.71** [.69, .73]			
4. UCLA Loneliness Scale-3	2.93	2.96	-.54** [-.57, -.51]	.56** [.53, .58]	.50** [.47, .53]		
5. SSS-8	4.68	4.87	-.59** [-.62, -.56]	.64** [.62, .66]	.59** [.56, .61]	.48** [.45, .51]	
6. EUROHIS-QOL-8	31.66	5.27	.68** [.66, .70]	-.62** [-.65, -.60]	-.52** [-.55, -.49]	-.51** [-.54, -.48]	-.59** [-.62, -.57]

Note. PHQ = Patient Health Questionnaire; GAD = Generalized Anxiety Disorder; UCLA = University of California, Los Angeles; SSS = Somatic Symptom Scale; EUROHIS-QOL = European Health Interview Survey - Quality of Life; Values in square brackets indicate the 95% confidence interval; * indicates p < .05., ** indicates p < .001.

Supplementary Table 9. Item-Level Correlations of the WHO-5 and depression symptoms (PHQ-2), anxiety symptoms (GAD-2), loneliness (UCLA Loneliness Scale-3), somatic symptoms (SSS-8), and quality of life (EUROHIS-QOL-8)

Item	PHQ-2	GAD-2	UCLA	SSS-8	EUROHIS-QOL
WHO-5					
cheerful and in good spirits (#1)	-0.55	-0.43	-0.51	-0.50	0.65
calm and relaxed (#2)	-0.55	-0.47	-0.50	-0.50	0.61
active and vigorous (#3)	-0.57	-0.41	-0.50	-0.55	0.65
woke up feeling fresh and rested (#4)	-0.57	-0.45	-0.47	-0.56	0.61
daily life filled with things that interest me (#5)	-0.57	-0.40	-0.51	-0.49	0.63

Note. PHQ = Patient Health Questionnaire; GAD = Generalized Anxiety Disorder; UCLA = University of California, Los Angeles; SSS = Somatic Symptom Scale; EUROHIS-QOL = European Health Interview Survey - Quality of Life; * indicates $p < .05$., ** indicates $p < .001$.

Supplementary Table 10. Latent correlations between the WHO-5 and depression symptoms (PHQ-2), anxiety symptoms (GAD-2), loneliness (UCLA Loneliness Scale-3), somatic symptoms (SSS-8), and quality of life (EUROHIS-QOL-8)

Model	Latent Correlation
PHQ-2	-0.74** [-0.78, -0.71]
GAD-2	-0.60** [-0.65, -0.55]
UCLA	-0.61** [-0.65, -0.57]
SSS-8	-0.65** [-0.68, -0.61]
EUROHIS-QOL	0.73** [0.70, 0.76]

Note. PHQ = Patient Health Questionnaire; GAD = Generalized Anxiety Disorder; UCLA = University of California, Los Angeles; SSS = Somatic Symptom Scale; EUROHIS-QOL = European Health Interview Survey - Quality of Life; Values in square brackets indicate the 95% confidence interval; * indicates $p < .05$., ** indicates $p < .001$.

Supplementary Table 11. Population based norms (cumulative percentiles) of the WHO-5 scores (male sample)

WHO-5	Total	age 16-24	age 25-34	age 35-44	age 45-54	age 55-64	age 65-74	age 75+
0	0.3<br>[0.08, 0.66]	< 0.1	< 0.1	0.7<br>[0, 2]	0.5<br>[0, 1.5]	0.8<br>[0, 1.9]	< 0.1	< 0.1
4	0.8<br>[0.41, 1.4]	< 0.1	< 0.1	1.4<br>[0, 3.4]	1<br>[0, 2.5]	1.1<br>[0, 2.6]	0.6<br>[0, 1.7]	2<br>[0, 5.1]
8	1.5<br>[0.82, 2.22]	< 0.1	< 0.1	1.4<br>[0, 3.4]	1<br>[0, 2.5]	2.6<br>[0.8, 4.5]	1.7<br>[0, 4]	4<br>[1, 8.1]
12	2.3<br>[1.48, 3.12]	< 0.1	< 0.1	1.4<br>[0, 3.4]	2<br>[0.5, 3.9]	3<br>[1.1, 4.9]	2.8<br>[0.6, 5.6]	9.1<br>[4, 16.2]
16	3.3<br>[2.3, 4.35]	< 0.1	1<br>[0, 2.5]	2.7<br>[0, 5.4]	3.4<br>[1, 5.9]	3.8<br>[1.5, 6]	4.5<br>[1.7, 7.9]	9.1<br>[4, 16.2]
20	5.8<br>[4.52, 7.31]	2.4<br>[0, 5.6]	2<br>[0.5, 4]	5.4<br>[2, 8.82]	4.4<br>[2, 7.4]	6<br>[3.4, 9]	9.6<br>[7.2, 13]	14.1<br>[8.1, 21.2]
24	7.3<br>[5.92, 8.79]]	2.4<br>[0, 5.6]	2.5<br>[0.5, 5]	7.4<br>[3.4, 12.2]	5.9<br>[2.9, 9.3]	8.3<br>[5.3, 11.7]	9.6<br>[5.6, 14.1]	19.2<br>[12.1, 27.3]
28	8.5<br>[6.9, 10.02]	2.4<br>[0, 5.6]	3.5<br>[1.5, 6.5]	7.4<br>[3.4, 12.2]	5.9<br>[2.9, 9.3]	12<br>[8.3, 15.8]	9.6<br>[5.6, 14.1]	21.2<br>[13.1, 29.3]
32	10.1<br>[8.3, 11.83]	4<br>[0.8, 7.3]	4.5<br>[2, 7.5]	9.5<br>[4.7, 14.9]	7.4<br>[3.9, 11.3]	12.4<br>[8.6, 16.5]	11.9<br>[7.3, 16.9]	26.3<br>[18.2,34 .33]
36	11.4<br>[9.61, 13.23]	4.8<br>[1.6, 8.9]	4.5<br>[2, 7.5]	10.1<br>[5.4, 15.5]	7.8<br>[4.4, 11.8]	13.9<br>[9.8, 18.4]	15.8<br>[10.7, 21.5]	28.3<br>[18.2, 34.33]
40	13.8<br>[11.91, 15.61]	5.6<br>[2.4, 9.7]	6<br>[3, 9.5]	11.5<br>[6.8, 16.9]	10.8<br>[6.4, 15.2]	16.2<br>[12, 21.1]	20.3<br>[14.7, 26]	31.3<br>[23.18, 40.4]
44	16.4<br>[11.91, 15.61]	6.5<br>[2.4, 11.3]	7<br>[3.5, 10.6]	13.5<br>[8.1, 18.9]	13.2<br>[8.79, 18.1]	19.5<br>[14.7, 24.4]	23.2<br>[16.9, 28.8]	37.4<br>[28.3, 46.5]
48	19.1<br>[16.76, 21.2]	10.5<br>[5.6, 16.1]	8.5<br>[5, 12.6]	17.6<br>[11.5, 23.6]	16.7<br>[11.8, 22.1]	21.8<br>[16.9, 27.1]	25.4<br>[19.2, 31.6]	39.4<br>[30.3, 49.5]
52	21.9<br>[19.47, 24.24]	12.9<br>[7.3, 19.4]	9.5<br>[6, 13.6]	18.9<br>[12.8, 25]	19.6<br>[14.2, 25]	25.2<br>[19.9, 30.1]	27.7<br>[20.9, 33.9]	47.5<br>[37.4, 57.6]
56	25.6<br>[23.17, 27.94]	16.9<br>[10.5, 23.4]	11.1<br>[7, 15.1]	23<br>[16.2, 30.4]	24.5<br>[18.59, 29.91]	28.6<br>[22.9, 33.81]	31.1<br>[24.3, 37.3]	53.5<br>[44.38, 62.6]
60	30.6	22.6	15.1	27	30.9	32.7	35.6	61.6

	[27.86, 33.28]	[16.1, 30.6]	[10.1, 20.1]	[19.6, 34.5]	[24, 36.8]	[26.7, 38.3]	[28.2, 42.4]	[44.38, 62.6]
64	34.6	25	17.6	33.1	33.3	36.8	39	71.7
	[31.88, 37.39]	[17.7, 33.1]	[12.6, 23.6]	[25.7, 40.5]	[26.5, 39.7]	[31.2, 42.5]	[31.6, 45.81]	[62.6, 79.8]
68	41.7	30.6	24.6	36.5	42.2	46.2	46.9	74.7
	[38.78, 44.62]	[23.4, 39.5]	[18.6, 31.2]	[28.4, 43.9]	[35.3, 48.5]	[40.6, 51.9]	[39, 53.7]	[66.7, 82.8]
72	47.9	39.5	30.2	41.9	45.1	54.1	56.5	76.8
	[45.03, 50.86]	[31.5, 48.4]	[24.1, 36.7]	[33.8, 50.02]	[38.19, 52]	[40.6, 51.9]	[48.6, 63.8]	[68.7, 83.8]
76	56.1	47.6	37.2	47.3	57.4	64.3	63.3	80.8
	[53.16, 58.83]	[39.5, 56.5]	[30.7, 44.2]	[39.2, 55.4]	[50.99, 64.2]	[58.6, 69.9]	[55.9, 70.11]	[72.7, 87.9]
80	76.3	65.3	64.8	73.6	77.9	82.7	80.8	88.9
	[73.79, 78.64]	[57.3, 74.2]	[58.3, 71.4]	[66.9, 81.1]	[71.6, 83.8]	[78.2, 87.2]	[74.6, 86.4]	[82.8, 94.9]
84	80.9	71	70.4	79.1	82.8	86.5	85.3	89.9
	[78.55, 82.99]	[62.88, 79]	[64.3, 76.9]	[72.3, 85.8]	[77.5, 87.7]	[82.3, 90.2]	[79.7, 90.4]	[83.8, 94.9]
88	85.5	79.8	75.9	84.5	86.8	90.2	88.1	93.9
	[83.4, 87.43]	[73.4, 86.3]	[70.4, 81.9]	[78.4, 90.5]	[82.39, 91.2]	[86.8, 93.6]	[83.59, 92.7]	[88.9, 98]
92	89.2	83.9	82.4	87.8	90.7	93.6	90.4	94.9
	[87.51, 90.88]	[77.4, 90.3]	[76.9, 87.4]	[82.39, 93.2]	[86.3, 94.6]	[90.6, 96.6]	[85.9, 94.4]	[89.9, 99]
96	90.6	84.7	83.4	87.8	93.1	95.9	91.5	94.9
	[88.82, 92.11]	[78.2, 90.32]	[78.39, 88.4]	[82.39, 93.2]	[89.2, 96.6]	[93.2, 98.1]	[87, 95.5]	[89.9, 99]
100	> 99.9	> 99.9	> 99.9	> 99.9	> 99.9	> 99.9	> 99.9	> 99.9

Note. All raw scores were multiplied by 4 in order to transform the original WHO-5 score range from 0–25 to a standardized scale of 0–100, as recommended in the scoring guidelines; Values in square brackets indicate the 95% confidence interval based on 1,000 bootstrap samples.

Supplementary Table 12. Population based norms (cumulative percentiles) of the WHO-5 scores (female sample)

WHO-5	Total	age 16-24	age 25-34	age 35-44	age 45-54	age 55-64	age 65-74	age 75+
0	0.2 [0.08, 0.54]	< 0.1	< 0.1	0.1	< 0.1	0.5 [0, 1.8]	< 0.1	1.4 [0, 3.6]
4	0.5 [0.15, 1]	< 0.1	< 0.1	0.9 [0, 2.3]	< 0.1	0.5 [0, 1.4]	< 0.1	2.9 [0.7, 5.8]
8	1.2 [0.69, 1.85]	< 0.1	1.1 [0, 2.61]	1.8 [0.5, 3.7]	< 0.1	0.5 [0, 1.41]	1 [0, 2.5]	5.1 [1.4, 8.72]
12	3.1 [2.24, 4.01]	4 [1, 7.9]	3.2 [1.1, 5.8]	2.3 [0.5, 4.6]	2.2 [0.4, 3.9]	1.8 [0.5, 3.6]	2.5 [0.5, 4.51]	8 [3.6,12.32]
16	4.4 [3.39, 5.48]	4 [1, 7.9]	4.7 [2.1, 7.91]	2.7 [0.9, 5]	3.1 [1.29, 5.2]	3.2 [1.4, 5.9]	4 [1.5, 7]	11.6 [6.5, 17.4]
20	7.8 [6.32, 9.25]	5 [1, 9.9]	6.8 [3.2, 10.5]	6.8 [3.7, 10.5]	6.1 [3.1, 9.2]	5.4 [2.7, 8.6]	9 [5, 13.6]	17.4 [11.6, 23.9]
24	8.6 [7.09, 10.18]	5 [1, 9.9]	7.9 [4.2, 12.1]	7.3 [4.1, 11.4]	7.4 [4.4, 10.9]	5.4 [2.7, 8.6]	10.6 [6.5, 15.1]	18.8 [12.98, 26.1]
28	10.5 [8.79, 12.34]	5.9 [2, 10.9]	8.9 [5.3, 13.7]	8.2 [4.6, 12.3]	9.2 [5.7, 13.1]	8.6 [5.4, 12.7]	11.1 [7, 15.1]	23.9 [17.4, 31.9]
32	12.4 [10.56, 14.26]	7.9 [3, 13.9]	11.1 [6.8, 15.8]	9.6 [5.9, 13.7]	10.5 [7, 14.4]	10 [6.3, 14.01]	13.1 [8.5, 18.1]	28.3 [21, 35.52]
36	14.3 [12.34, 16.19]	8.9 [4, 14.9]	12.6 [8.4, 17.4]	11.4 [7.3, 16]	10.9 [7.4, 14.8]	11.3 [7.7, 15.8]	16.1 [11.1, 21.6]	32.6 [25.38, 40.6]
40	17.4 [15.42, 19.58]	13.9 [7.9, 21.8]	13.7 [8.9, 18.4]	13.2 [9.1, 17.8]	13.1 [9.2, 17.5]	14.9 [10.9, 19.9]	20.1 [14.6, 26.1]	39.1 [31.2, 47.8]
44	20.3 [18.12, 22.51]	18.8 [11.9, 26.7]	15.8 [11.1, 21.1]	15.5 [11, 20.1]	16.2 [11.8, 20.5]	17.6 [13.1, 22.6]	23.1 [17.6, 29.1]	42 [34.1, 50.7]
48	23.4 [21.2, 25.52]	21.8 [13.9, 30.7]	20 [14.2, 25.8]	18.3 [13.2, 23.7]	17.9 [13.1, 22.7]	19.5 [14.5,24.41]	29.1 [23.1, 35.7]	44.2 [35.5, 52.9]
52	25.9 [23.67, 28.22]	22.8 [14.9, 31.7]	21.6 [15.8, 27.4]	23.3 [17.8, 28.81]	19.7 [14.8, 24.9]	21.3 [16.3, 27.1]	30.7 [24.6, 37.7]	49.3 [40.6, 58]
56	28.6 [26.29, 30.92]	28.7 [19.8, 37.6]	24.2 [18.4, 30]	24.7 [18.7, 30.6]	23.6 [18.8, 29.3]	24.4 [19.49, 30.3]	31.7 [25.6, 38.7]	51.4 [42.8, 60.1]
60	34.1 [31.46, 36.62]	34.7 [25.7, 44.6]	25.8 [19.99, 32.1]	29.7 [23.7, 36.1]	28.8 [23.6, 34.5]	32.1 [26.2, 38.5]	38.2 [32.2, 44.7]	58 [49.3, 65.9]

64	37.2<br>[34.69, 39.86]	36.6<br>[27.7, 46.5]	28.4<br>[22.1, 34.71]	32.9<br>[26.9, 39.3]	31.9<br>[26.2, 37.61]	36.2<br>[29.9, 43]	42.7<br>[35.7, 49.7]	59.4<br>[51.4, 67.4]
68	43.1<br>[40.25, 45.8]	38.6<br>[28.7, 48.5]	32.6<br>[26.3, 39.5]	39.7<br>[33.3, 46.11]	36.7<br>[30.1, 42.8]	42.1<br>[35.69, 48.9]	49.7<br>[43.19, 56.8]	68.8<br>[61.6, 76.8]
72	50.9<br>[48.03, 53.66]	41.6<br>[31.7, 51.5]	37.9<br>[31.1, 44.7]	49.3<br>[42.89, 55.7]	46.7<br>[39.69, 53.3]	51.6<br>[44.79, 58.4]	57.3<br>[50.28, 64.31]	74.6<br>[67.4, 81.9]
76	58.7<br>[55.9, 61.37]	46.5<br>[36.6, 56.4]	46.3<br>[39.5, 53.2]	55.7<br>[48.9, 62.1]	55<br>[48.5, 61.1]	62<br>[55.2, 68.3]	67.8<br>[50.28, 64.31]	76.8<br>[69.6, 84.1]
80	79<br>[76.72, 81.11]	60.4<br>[50.5, 70.3]	71.6<br>[65.3, 77.9]	78.1<br>[72.6, 83.6]	81.2<br>[76, 86.5]	81<br>[75.1, 86]	82.4<br>[76.89, 87.4]	92.8<br>[88.4, 97.1]
84	83.3<br>[81.19, 85.12]	72.3<br>[63.4, 80.2]	76.8<br>[70.5, 82.6]	82.2<br>[76.7, 87.2]	83.4<br>[78.2, 87.8]	86.4<br>[81.4, 90.5]	85.9<br>[80.9, 90.5]	92.8<br>[88.4, 97.1]
88	88.1<br>[86.35, 89.9]	84.2<br>[76.2, 90.1]	82.6<br>[77.4, 88.4]	84.9<br>[79.9, 89.5]	88.2<br>[84.29, 92.1]	91.4<br>[87.3, 94.6]	90.5<br>[85.9, 94.5]	94.9<br>[91.3, 98.6]
92	91.8<br>[90.29, 93.22]	89.1<br>[82.2, 95]	91.1<br>[86.8, 95.3]	89<br>[84.9, 92.7]	92.1<br>[88.59, 95.2]	93.2<br>[89.6, 96.4]	92.5<br>[88.39, 96]	94.9<br>[91.3, 98.6]
96	93.4<br>[92.06, 94.83]	90.1<br>[84.2, 95]	91.6<br>[87.4, 95.3]	92.2<br>[88.6, 95.4]	93.4<br>[90, 96.5]	94.1<br>[91, 96.8]	95.5<br>[92.5, 98.5]	96.4<br>[92.8, 99.3]
100	>99.9	>99.9	>99.9	>99.9	>99.9	>99.9	>99.9	>99.9

Note. All raw scores were multiplied by 4 in order to transform the original WHO-5 score range from 0–25 to a standardized scale of 0–100, as recommended in the scoring guidelines; Values in square brackets indicate the 95% confidence interval based on 1,000 bootstrap samples.

.

Supplementary Table 13. Results of measurement invariance analyses across gender, age, and their interaction (age × gender)

	χ2	*df*	CFI	ΔCFI	RMSEA	ΔRMSEA	Measurement invariance
Gender (male, female)							
Configural invariance	105.129	10	1	-	0.087	-	-
Threshold invariance[1]	112.172	25	1	0	0.053	-0.034	✓
Metric invariance[2]	115.174	29	1	0	0.049	-0.003	✓
Scalar invariance[3]	122.442	33	1	0	0.046	-0.002	✓
Full invariance[4]	133.433	38	1	0	0.045	-0.002	✓
Age							
Configural invariance	96.781	10	1	-	0.083	-	-
Threshold invariance[1]	104.812	25	1	0	0.05	-0.033	✓
Metric invariance[2]	107.776	29	1	0	0.046	-0.004	✓
Scalar invariance[3]	122.235	33	1	0	0.046	0	✓
Full invariance[4]	144.202	38	0.999	0	0.047	0.001	✓
Age x Gender							
Configural invariance	119.186	20	0.999	-	0.089	-	-
Threshold invariance[1]	142.972	65	1	0	0.044	-0.045	✓
Metric invariance[2]	149.852	77	1	0	0.039	-0.005	✓
Scalar invariance[3]	173.223	89	1	0	0.039	0	✓
Full invariance[4]	205.28	104	0.999	0	0.039	0.001	✓

Note. All fit statistics are robust; CFI = Comparative Fit Index; CFI = CFI-differences for the different measurement invariance levels; RMSEA = Root Mean Square Error of Approximation; RMSEA = RMSEA- differences for the different measurement invariance levels; ✓= ΔCFI < -.010 complemented by RMSEA ≥ .015 indicates a violation of measurement invariance; marks measurement invariance for the respective level; [1] equivalency of thresholds; [2] equivalency of thresholds + factor loadings; [3] equivalency of thresholds + factor loadings + equivalency of constants; [4] equivalency of thresholds + factor loadings + equivalency of constants + unique-factor variance.

Supplementary Table 14. Results of measurement invariance analyses regarding anxiety (GAD-2), depression (PHQ-2), and somatic symptoms (SSS-8)

	χ2	*df*	CFI	ΔCFI	RMSEA	ΔRMSEA	Measurement invariance
Anxiety (no, yes)							
Configural invariance	82.993	10	0.999	-	0.123	-	-
Threshold invariance[1]	113.929	24	0.999	0	0.078	-0.045	✓
Metric invariance[2]	126.7	28	0.999	0	0.071	-0.007	✓
Scalar invariance[3]	133.167	32	0.999	0	0.065	-0.005	✓
Full invariance[4]	158.849	37	0.999	0	0.065	-0.001	✓
Depression (no, yes)							
Configural invariance	70.741	10	1	-	0.119	-	-
Threshold invariance[1]	95.172	24	1	0	0.072	-0.047	✓
Metric invariance[2]	103.363	28	1	0	0.065	-0.007	✓
Scalar invariance[3]	106.998	32	1	0	0.061	-0.004	✓
Full invariance[4]	127.211	37	0.999	0	0.059	-0.002	✓
Somatization (Minimal/Low, Medium/High)							
Configural invariance	96.347	10	0.999	-	0.143	-	-
Threshold invariance[1]	116.499	25	0.999	0	0.075	-0.068	✓
Metric invariance[2]	117.447	29	0.999	0	0.068	-0.007	✓
Scalar invariance[3]	154.092	33	0.999	0	0.072	0.005	✓
Full invariance[4]	215.527	38	0.999	0	0.079	0.007	✓

Note. All fit statistics are robust; CFI = Comparative Fit Index; CFI = CFI-differences for the different measurement invariance levels; RMSEA = Root Mean Square Error of Approximation; RMSEA = RMSEA- differences for the different measurement invariance levels; ✓= ΔCFI < - .010 complemented by RMSEA ≥ .015 indicates a violation of measurement invariance; marks measurement invariance for the respective level; [1] equivalency of thresholds; [2] equivalency of thresholds + factor loadings; [3] equivalency of thresholds + factor loadings + equivalency of constants; [4] equivalency of thresholds + factor loadings + equivalency of constants + unique-factor variances.
